# Repurposing the Antidiabetic Drugs Glyburide, Gliquidone, and Glipizide in Combination with Benznidazole for *Trypanosoma cruzi* Infection

**DOI:** 10.3390/ph18010021

**Published:** 2024-12-27

**Authors:** Citlali Vázquez, Rusely Encalada, Isabel Jiménez-Galicia, Rogelio Gómez-Escobedo, Gildardo Rivera, Benjamín Nogueda-Torres, Emma Saavedra

**Affiliations:** 1 Department of Biochemistry, Instituto Nacional de Cardiología Ignacio Chávez, Mexico City 14080, Mexico; cita2812@gmail.com (C.V.); eencaladarusely@yahoo.com.mx (R.E.); galbel.2610@gmail.com (I.J.-G.); 2 Departamento de Parasitología, Escuela Nacional de Ciencias Biológicas, Instituto Politécnico Nacional, Mexico City 11340, Mexico; rogelio.gomez.14@gmail.com (R.G.-E.); bnogueda@yahoo.com (B.N.-T.); 3 Laboratorio de Biotecnología Farmacéutica, Centro de Biotecnología Genómica, Instituto Politécnico Nacional, Reynosa 88710, Mexico; giriveras@ipn.mx

**Keywords:** Chagas disease, drug repurposing, antidiabetic drug, hypoglycemic drug, benznidazol, combination therapy

## Abstract

Infection with the protozoan parasite *Trypanosoma cruzi* causes human Chagas disease. Benznidazole (BNZ) and nifurtimox are the current drugs for the treatment; however, they induce severe adverse side effects in patients; therefore, there is a need to improve the treatment effectiveness and efficiency of these drugs for its safer use. **Background/Objective**: Glyburide, glipizide, and gliquidone, hypoglycemic drugs for diabetes treatment, were previously predicted to bind to dihydrofolate reductase-thymidylate synthase from *T. cruzi* by in silico docking analysis; they also showed antiproliferative effects against *T. cruzi* epimastigotes, the stage of the insect vector. In the present study, the potential parasiticidal effect of these antidiabetic drugs was tested in monotherapy and bi-therapy with BNZ in human cells in vitro and in animals. **Methods**: Evaluation was performed in (a) a model of in vitro infection of *T. cruzi* trypomastigotes using human fibroblasts as host cells and (b) in mice infected with *T. cruzi*. **Results:** The antidiabetic drugs in monotherapy showed antiparasitic effects in preventing infection progression (trypomastigotes release), with an IC_50_ of 8.4–14.3 µM in comparison to that of BNZ (0.26 µM) in vitro. However, in bi-therapy, the presence of just 0.5 or 1 µM of the antidiabetics decreased the BNZ IC_50_ by 5–10 times to 0.03–0.05 µM. Remarkably, the antidiabetic drugs in monotherapy decreased the infection in mice by 40–60% in a similar extent to BNZ (80%). In addition, the combination of BNZ plus antidiabetics perturbed the antioxidant metabolites in epimastigotes. **Conclusions**: These results identified antidiabetics as potential drugs in combination therapy with BNZ to treat *T. cruzi* infection.

## 1. Introduction

Chagas disease, or American trypanosomiasis, in humans is caused by the infection with the trypanosomatid protozoan parasite *Trypanosoma cruzi* (*T. cruzi*) [1,2]. The World Health Organization (WHO) estimates that 7 million people worldwide are infected with the parasite, mainly in Latin American countries, but that 75 million people are at risk of being infected because of living in endemic regions [3]. Furthermore, cases in non-endemic countries in Europe, Asia, Oceania, the USA, and Canada have been reported in immigrant persons. The disease causes around 12,000 deaths annually [3].

The parasite infection initiates by the invasion of skin breaches or mucosa by the trypomastigotes eliminated in the feces or urine of the triatomine vector during the blood feeding, or by organ transplantation and blood transfusion from infected persons, congenital transmission, laboratory accidents, or ingestion of food contaminated with the parasite [4].

The disease progression presents two stages. The acute one, which lasts approximately two months, is characterized by high levels of blood parasitemia without or with mild and unspecific symptoms after parasite infection. The chronic stage can be developed throughout several years after the primary infection, which may occur unnoticed; however, 30–40% of the patients display severe cardiomyopathies and 10% of patients develop inflammation of the gastrointestinal tract (megasyndrome) [1,2] and denervation, particularly from the parasympathetic nervous system [5]. In this stage, cardiac arrhythmias or insufficiency, progressive heart failure, or sudden death occur due to destruction of the innervation of the cardiac muscle [2,5,6].

**Scheme 1:**
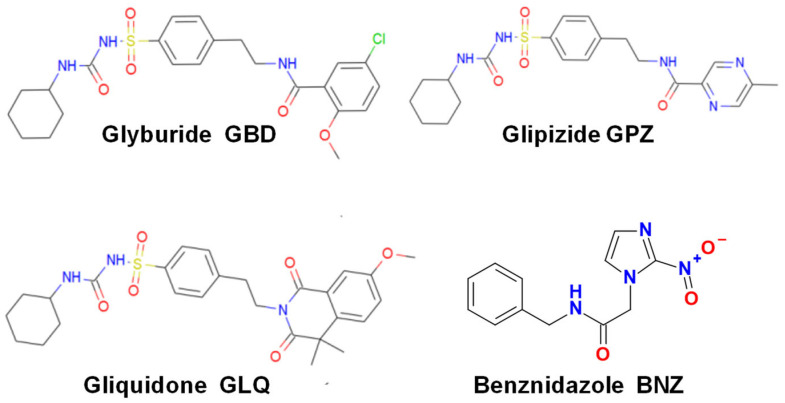
Chemical structures of the canonic antichagasic BNZ and the antidiabetic drugs used in the present study.

Benznidazol (BNZ) (Scheme 1) and nifurtimox (Nfx) are recognized by the WHO as efficacious drugs for the treatment of *T. cruzi* infection, mainly in the acute phase or in early chronic infections [1,3,7,8]. BNZ is preferred over Nfx due to better tolerability and tissue penetration and a lower rate of treatment interruption (9–29%) than nifurtimox (14–75%) [1,7,8]. Adverse side effects have been described for both antichagasic drugs; for BNZ they include skin hypersensibility, edema, muscle and joint pain, and polyneuropathy [9], whereas the most common Nfx side effects are anorexia, weight loss, neurological disorders, nausea, and vomiting [10]. Both drugs are effective for treatment in the acute and early chronic stage phases, showing fewer and moderate adverse effects in children; however, the adverse effects in adults are severe. Moreover, their use in the advanced chronic stage is limited by the health frailty of these patients [11]. For these reasons, new antichagasic treatment strategies with better efficacy and safety are necessary.

In recent years, repurposing of drugs of common medical use to treat infectious diseases for which they were not originally designed is a promising clinical alternative, which also saves costs and time in identifying new antiparasitic drugs [12,13,14]. By applying classical docking analysis and protein–ligand interaction profiling of an FDA-approved drug library against the dihydrofolate reductase-thymidylate synthase of *T. cruzi*, it was predicted that the antidiabetic compounds glipizide (GPZ), gliquidone (GLQ), and glyburide (GBD; also known as glybenclamide) (Scheme 1) are potent and specific enzyme inhibitors [15]. These compounds are ATP-sensitive potassium channel inhibitors of the sulfonylurea type that promote insulin secretion for the treatment of type-2 diabetic patients [16,17,18]. When tested against epimastigotes they showed antiproliferative effects, with an IC_50_ of 13.4, 12, and 66 µM, for GPZ, GLQ, and GBD, respectively [15].

Previous studies on the use of GBD against *Leishmania* infection showed that the antidiabetic alone decreased the infection and increased glucantime efficacy in a synergic fashion [19,20,21,22]. It was also predicted by in silico docking analysis that GBD can bind to *Leishmania* trypanothione synthetase (TryS) with high affinity [23]. TryS is the enzyme responsible for trypanothione [T(SH)_2_] synthesis; the latter is the specific and main antioxidant metabolite in trypanosomatids, and its related enzymes and metabolism are sites for therapeutic intervention [24,25,26]. Thus, the sulfonylurea-type antidiabetic drugs seem to have potent non-canonical inhibitory effects on the trypanosomatid metabolism and cell functions; therefore, it was foreseen that GBD, GPZ, and GLQ may have potent and specific effects on the parasite, and hence, they might be used for the treatment of *T. cruzi* infection.

In this study the effect of GBD, GPZ, and GLQ on preventing the progression of the infection with *T. cruzi* in a model of in vitro infection of human fibroblasts was evaluated in monotherapy and bi-therapy with BNZ. Drug combination therapy may bring about the use of lower doses to thus attenuate the severe side effects of the canonical BNZ treatment. Furthermore, the effect of the antidiabetic drugs on decreasing parasitemia in the *T. cruzi* acute infection in mice was also analyzed. It was also explored whether treatment with the hypoglycemic compounds alters the thiol pools in *T. cruzi*.

## 2. Results

### 2.1. Effect of BNZ and Antidiabetics in Monotherapy

To evaluate the effects of the antidiabetics alone, a model of in vitro infection with trypomastigotes of monolayers of human foreskin fibroblasts 1 (HFF-1) was used as described in the methodology; the criterium to determine efficacy was the ability to decrease the trypomastigotes’ release after the 4th–5th day post-infection. The *T. cruzi* Querétaro strain (TBAR/MX/0000/Querétaro) was used, which is classified in the Tc1 group [27], and is characterized by its high degree of virulence [28].

**Figure 1:**
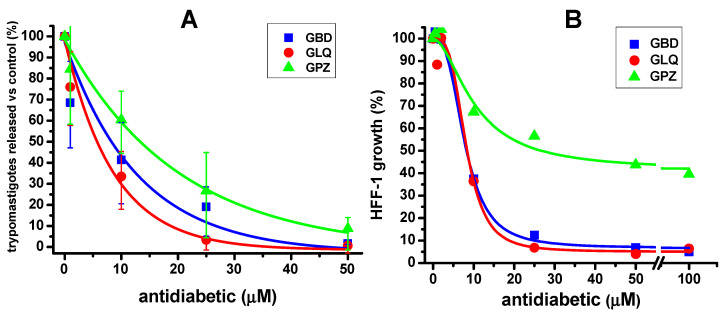
Effect of antidiabetic drugs in monotherapy in a model of in vitro *T. cruzi* Querétaro strain infection. (**A**) The trypomastigotes burst after 5 days of in vitro infection in the presence of increasing concentrations of the antidiabetics is shown. In (**B**), the effect of the antidiabetics on cell host growth (HFF-1, human foreskin fibroblasts 1) after 5 days is shown. The experimental data points are the average and standard deviation of the number of independent experiments, which was n = 4 in (**A**) and n = 2 in (**B**). In (**A**), 100% trypomastigotes release in the absence of any drug (0 µM) was 1 ± 0.4 × 10^6^ parasites/mL; in (**B**), 100% cell growth was 1.33 ± 0.0057 × 10^5^ cells/mL. Data were fitted to the dose–response equation using Origin 8 Software.

Figure 1 shows the effect of the antidiabetic drugs in monotherapy on the liberation of trypomastigotes, which were effective at 10 µM or lower concentrations (Figure 1A, Table 1). As a control, the cell growth of HFF-1 host cells exposed for five days to the antidiabetic drugs was determined (Figure 1B, Table 1), showing 50% decrease in growth at 5–10 µM for GBD and GLQ, and 35 µM for GPZ. These latter results suggested that cell growth inhibition might partially contribute to the effect of GBD and GLQ on the release of trypomastigotes at concentrations > 5 µM.

### 2.2. Effect of BNZ and Antidiabetics in Bi-Therapy

**Figure 2:**
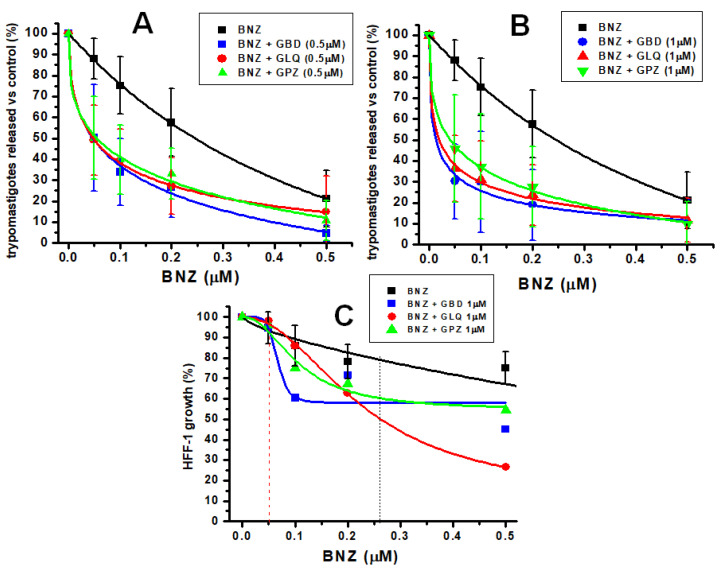
Effect of BNZ alone or in bi-therapy with antidiabetics on *T. cruzi* Queretaro strain in vitro infection and on HFF-1 growth. Parasite burst after 5 days of in vitro infection in the presence of variable BNZ concentrations (0 to 0.5 µM) plus fixed 0.5 µM (**A**) or 1 µM (**B**) of the antidiabetics. In (**C**), the effect of variable BNZ concentrations in the absence or presence of 1 µM of antidiabetics on HFF-1 host growth after 5 days is shown. In all the experiments, 0 means no drug added. The experimental data shown represent the mean and standard deviation of the number of independent experiments, which was n = 8 for (**A**,**B**), and for (**C**), n = 3 for BNZ in monotherapy and n = 2 for bi-therapy. The 100% trypomastigote release in the absence of any drug (point 0) was 1 ± 0.4 × 10^6^ parasites/mL. The 100% cell growth in the absence of any drug was 8.4 ± 3.4 × 10^4^ cells/mL. Data in (**A**,**B**) were fitted to the dose–response equation; in (**C**), data were fitted to the logistic equation (with no mechanistic meaning) using Origin 8.0 software.

It was further evaluated whether the antidiabetic drugs could decrease the IC_50_ of BNZ for trypomastigotes bursting (Figure 2 and Table 1). BNZ alone potently decreased the release of trypomastigotes at concentrations up to 0.5 µM, with an IC_50_ of 0.26 µM (Figure 2A,B). Then, the IC_50_ of BNZ was determined in the presence of fixed low doses of 0.5 µM and 1 µM of the antidiabetics; these concentrations were selected in which the cell viability and growth of HFF-1 cells were not compromised (Figure 1B). The results indicate that the presence of the antidiabetics at low concentrations significantly decreased the IC_50_ of BNZ by 5.2–8.6 folds, to 0.05 µM or lower values (Figure 2A,B; Table 1). For instance, the 40% inhibition of infection by 0.2 µM BNZ alone (Figure 2A) was significantly increased up to 70% (Figure 2A) or 80% (Figure 2B) when combined with 0.5 or 1 µM GBD, respectively.

As a rigorous control, the growth of HFF-1 cells in bi-therapy was also evaluated. BNZ in monotherapy and bi-therapy with antidiabetic showed cytostatic effects (Figure 2C); at the IC_50_ of BNZ inhibition on release of trypomastigotes in monotherapy (0.26 µM), BNZ decreased the growth of host cells by 20%, whereas in bi-therapy with 1µM antidiabetics, the inhibition increased to 40–50% (Figure 2C black vertical dotted line). However, at the lowered IC_50_ values of BNZ (0.05 µM) for trypomastigote release in bi-therapy with 1 µM of the antidiabetics, HFF-1 growth was not affected (Figure 2C, red vertical dashed line). These results clearly indicate that the combination of BNZ with low doses of the antidiabetic lowered the BNZ concentration necessary to decrease infection, displacing it to a safer range where no adverse side effects on host cells are promoted. Table 1 summarizes the IC_50_ of BNZ and antidiabetics in monotherapy and bi-therapy.

### 2.3. Effect of the Antidiabetic Drugs on an Acute Model of T. cruzi Infection in Mice

**Figure 3:**
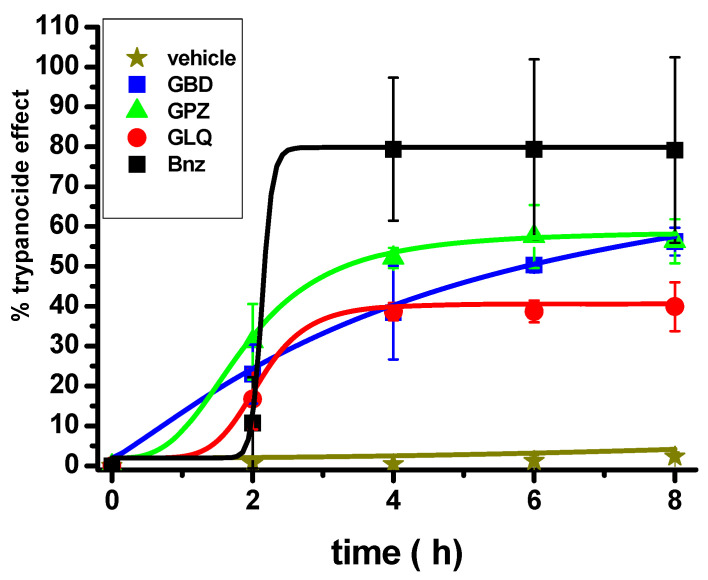
Effect of BNZ and antidiabetic drugs in monotherapy on *T. cruzi* Ninoa strain infection in a murine model. After parasitemia was established in the animals (12–18 days post inoculation), they were treated at a single dose of 100 mg/kg weight of BNZ, and antidiabetics and trypomastigotes were counted every 2 h in animals’ blood samples. The vehicle was Arabic gum. The number of mice was 3 for each group. Kruskall–Wallis non parametric analysis was *p* ≤ 0.01 for BNZ, GBD, and GPZ versus vehicle; *p* ≤ 0.01 for BNZ versus GLQ.

The effect of the antidiabetic drugs and BNZ in the clearance of blood trypomastigotes in mice infected with *T. cruzi* in acute drug treatment in monotherapy was evaluated. *Trypanosoma cruzi* Ninoa strain was used for these experiments; this strain is classified in the Tc1 group like the Querétaro strain [27]; Ninoa is characterized by lower virulence and therefore lower mortality in mice compared to the Querétaro strain [28]. However, epimastigotes of Ninoa strain are more resistant to BNZ [27]. Then, the *T. cruzi* Ninoa strain seems to be a more appropriate parasitic source to evaluate drug susceptibility in mice. The infected animals were treated at the peak of parasitemia with the drugs at a single dose of 100 mg drug/kg of weight, which is 15–30 folds lower than the lethal doses in mice (1500–3000 mg/kg) [29,30], and the presence of blood trypomastigotes was monitored every 2 h up to 8 h, which is the interval of the half-life of the antidiabetics in humans [17]. BNZ alone efficiently decreased the infection by 80% (Figure 3). GBD and GPZ alone also decreased the parasite infection by 60% in a significant manner with respect to the vehicle and similar to BNZ, whereas GLQ showed a lower effect than BNZ (*p* ≤ 0.01); the Kruskal–Wallis statistical test allowed these significant differences to be determined. These results indicate that GBD and GPZ were effective for decreasing *T. cruzi* parasitemia in mice. It is expected that combining BNZ with the antidiabetics may bring about an increased, synergistic inhibitory effect on infection, but this will require further implementation of lengthy treatments and re-dosing of the drugs at lower concentrations.

### 2.4. Effect on Thiol Metabolites

**Scheme 2:**
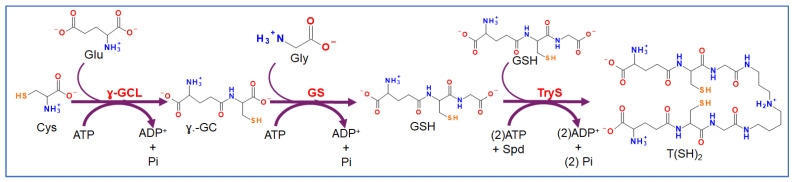
Trypanothione synthesis pathway in trypanosomatids. Trypanothione [T(SH)_2_] is synthesized from two molecules of glutathione (GSH) and one of spermidine (Spd) in a reaction catalyzed by trypanothione synthetase (TryS). In turn, GSH is synthesized from cysteine (Cys), glutamate (Glu), and glycine (Gly) by gamma-glutamylcysteine ligase (γ-GCL) and glutathione synthetase (GS) with intermediate gamma-glutamylcysteine (γ-GC). ATP is used in the three reactions.

The effect of monotherapy and bi-therapy in the content of thiol metabolites cysteine (Cys), glutathione (GSH), and trypanothione [T(SH)_2_] was evaluated. This was done in epimastigotes due to the high amount of biological material necessary for thiol determination. The precursors for T(SH)_2_ synthesis are Cys, GSH, and spermidine (Scheme 2). We have previously demonstrated that Cys but not spermidine is the limiting precursor for T(SH)_2_ synthesis [25,31,32]; hence, the analyses were focused on thiol metabolites.

**Figure 4:**
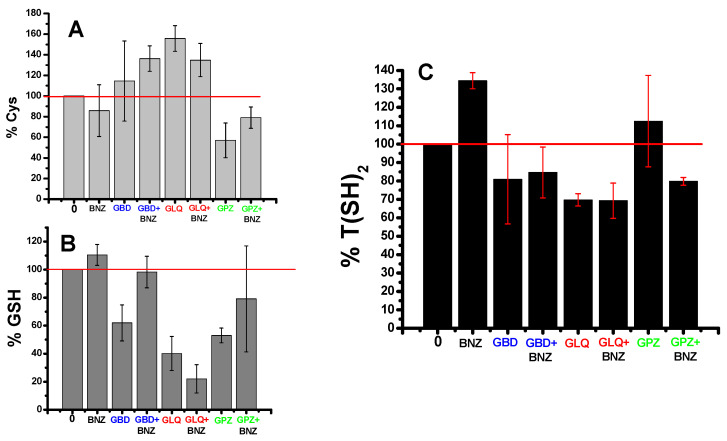
Effect of BNZ and antidiabetics in mono- and bi- therapy on thiol metabolites of *T. cruzi* Querétaro strain epimastigotes. Epimastigotes were treated for 24 h with 1 µM of BNZ and 1 µM antidiabetics alone or in combination. Afterwards, thiol metabolites Cys (**A**), GSH (**B**) and T(SH)_2_ (**C**) were determined by high-performance liquid chromatography (HPLC). Data are the mean ± standard deviation of three independent experiments. The 100% metabolite contents (horizontal red line) correspond to Cys = 5.1 ± 1.5, GSH = 5.6 ± 3.4, and T(SH)_2_ = 5.8 ± 2.4 nmol/mg cell protein from untreated epimastigotes.

BNZ seems to slightly decrease the Cys level for GSH and T(SH)_2_ synthesis (Figure 4). GBD and GLQ alone decreased the levels of GSH and T(SH)_2_ and increased those of Cys, suggesting inhibition at the beginning of the pathway. Interestingly, GPZ alone increased the level of T(SH)_2_ and decreased the levels of Cys and GSH; however, the increase in T(SH)_2_ cannot explain the near 50% decrease in Cys and GSH. Remarkably, the combination of BNZ with any of the antidiabetics decreased the T(SH)_2_ levels (Figure 4C). The changes in these antioxidant metabolites suggested perturbation of the antioxidant system in the parasite.

## 3. Discussion

### 3.1. Effectiveness of BNZ Alone and in Combination with Antidiabetics

There is an urgent need to develop new therapeutic strategies or to improve the existing ones for the treatment of human infection with *T. cruzi*. BNZ is an effective drug to diminish or eliminate the infection of the parasite in infected people that is early and promptly diagnosed; however, more treatment difficulties are encountered for patients in the chronic stage [2,33,34,35]. The severe adverse side effects become a factor of concern when deciding on treatments for the infected chronic patient [1,2,7,9,10,11].

Drug repurposing or repositioning appears as a promising strategy to identify new potentially successful antichagasic drugs, thus also saving time and costs. Computationally aided methods on selected targets [36,37] and, recently, in silico screening of FDA-approved drugs on parasite proteins and enzymes have emerged as innovative and promising approaches to repurposing drugs [13,14]. Using such approaches, we previously identified GLB, GLQ, and GPZ as potent inhibitors of *T. cruzi* epimastigotes growth [15]. The antidiabetics alone showed lower or similar efficacy than BNZ in epimastigotes cultured in vitro. The IC_50_ values on the growth of epimastigotes were 66, 12, and 13.4 µM for GBD, GLQ, and GPZ, respectively [15], compared to the IC_50_ of BNZ of 12 ± 2 µM [25] or 9.4 ± 1.3 µM [38]. It is worth noting that both BNZ IC_50_ determinations were attained under the same experimental conditions and are similar [25,38], and they are comparable to those reported by other research groups in other strains [39,40]. Furthermore, trypomastigotes isolated from infected cells and exposed in vitro for 3 h to BNZ (before transformation into amastigotes) showed a lethal concentration 50 (LC_50_) to 183 µM BNZ [38].

Here, the effect of BNZ and the antidiabetic drugs on in vitro infection of human mammalian cells by *T. cruzi* was explored. Although more laborious experimentally than drug evaluation with epimastigotes, the experimental approach used here provided a reliable and robust setup for proof-of-concept evaluation before its testing in animal models. Remarkably, BNZ required an extremely low concentration (0.26 µM, a lower dose than for epimastigotes) to decrease the in vitro cell infection, demonstrating its high efficacy. The antidiabetics alone did not compete with BNZ in its anti-infective effect, requiring up to 30-fold higher concentrations than that of BNZ to attain the same effect. However, the presence of 0.5 µM or 1 µM antidiabetics (which were innocuous on host cell growth) was able to significantly lower the IC_50_ of BNZ by 5–8 times. These results suggest that the combination of the antidiabetics can decrease the doses of BNZ required to cure the infection and support continued investigation in animal infection models.

Evaluation of the antidiabetic drugs in monotherapy in animals infected with *T. cruzi* produced encouraging results, showing a parasiticidal effect in an acute treatment and short time monitoring of parasitemia (8 h). Although BNZ was still the most effective drug to decrease the infection in the animals, the antidiabetics also showed parasiticidal effects; particularly, GBD and GPZ showed potent effects and seemed less toxic than GLQ (Figure 2C). This observation suggests that the antidiabetics may be considered as alternative antichagasic drugs to be used in combination with BNZ. It should be pointed out that the plasma concentrations of these drugs (sulfonylureas) that exert antidiabetic effects is in the range of 50–200 nM [18,41], which can be achieved with very low oral drug doses (<5 mg/day or 10 µmol GBD/day). The present results support further studies on combination therapies with BNZ in in vivo assays, both in the long and short term, in order to search for the decrease in the adverse effects of BNZ and, in addition, to determine the modes of action to decrease or eliminate *T. cruzi* parasitemia.

The *T. cruzi* Ninoa strain was used for the in vivo model evaluation because there were no in vivo drug-treating studies with the Queretaro strain to make comparisons with the results of the present study, as there are for the Ninoa strain [42,43]. Furthermore, epimastigotes of the *T. cruzi* Ninoa strain are more resistant to BNZ than those from the Querétaro and even CLBrener strains (IC_50_ on growth are 8.21, 4.29, and 5.54 µM, respectively [27]). Therefore, it seems of greater clinical interest to carry out the in vivo studies on a parasite strain with higher BNZ resistance.

It remains to be determined in animal models the effect of the bi-therapy BNZ + antidiabetics in short-acute and long-duration treatment protocols followed by post-treatment evaluation with several parasitological and serological tests that effectively demonstrate the elimination of the infection and its null or negligible effect on the physiological functions of the animals.

To our knowledge, this is the first time that antidiabetic drugs of the sulfonylurea type have been repurposed to treat *T. cruzi* infection. New-generation, safer hypoglycemic drugs such as glimepiride and glycazide have been developed [44] that should also be tested. These results open a possibility for combination therapy with lower doses of BNZ and antidiabetics. On the other hand, recently, metformin, another hypoglycemic compound of the biguanide family with a different mode of action than sulfonylurea, has shown biological effects against *Echinococcus* [45], *Toxoplasma* [46], and *Acanthamoeba* [47] parasites.

### 3.2. Possible Mode of Action

The most known and studied effect of the sulfonylurea antidiabetics is the inhibition of the ATP-gated potassium channels in pancreatic beta cells. This inhibition induces intracellular potassium build-up, which causes depolarization of the plasma membrane and promotes opening of the calcium channels; the ensuing increase in cytosolic calcium induces insulin secretion [16,17,18]. The previously reported anti-infective effect of GBD (glibenclamide) in *Leishmania* has been suggested to be through inhibition of ATP-binding cassette MDR (multidrug resistance) transporters; these transporters mediate the sequestration of glucantime into intracellular organelles, hence their inhibition by GBD could promote increasing the drug intracellularly [19,20,21,22]. In this regard, it has been proposed that GBD decreased the glucose uptake in GBD-resistant *Leishmania* [48]. GBD has also shown anti-helmintic activity on *Echinococcus granulosus* infection in mice in monotherapy, although there was no synergistic effectiveness in the bi-therapy with albendazole [49].

On the other hand, a previous report of in silico docking analysis in *Leishmania* TryS suggested that the enzyme can bind GBD; further, *Leishmania donovani* promastigotes exposed to GBD decreased their growth [23]; however, a direct relationship with inhibition of the antioxidant defense was not experimentally demonstrated. Here, epimastigotes exposed to antidiabetics alone or in combination with BNZ showed a significant decrease in T(SH)_2_, an effect that was not observed with BNZ alone (Figure 4). It is noted that the GBD and GLQ concentration used was 1 µM, which was much lower than the IC_50_ value on epimastigotes growth for the compounds in monotherapy (66 and 12 µM, respectively) [15]. GPZ decreased T(SH)_2_ only in combination with BNZ. These results suggest that antidiabetics can perturb the antioxidant capacity of the parasite, which is necessary to contend with the oxidative stress during host cell infection.

## 4. Materials and Methods

### 4.1. Cell Culture

Epimastigotes of the *T. cruzi* Queretaro strain (TBAR/MX/0000/Queretaro) [27,50] were cultured at 28 °C in LIT medium (liver infusion-tryptose: 0.5% tryptose, 0.5% liver infusion, 0.2% glucose, 0.4% NaCl, 0.04% KCl, 0.42% Na_2_HPO_4_) supplemented with 10% fetal bovine serum FBS (Biowest; Nuaillie, France); antibiotic mixture, 100 U penicillin/mL and 100 µg streptomycin/mL (Sigma-Aldrich, Darmstadt, Germany) and 25 µg/mL hemin (Santa Cruz; Dallas, TX, USA). The parasites were harvested at the exponential growth phase (4 days) by centrifugation at 2630× *g* and washed with phosphate-buffered saline pH 7.4 (PBS: 137 mM NaCl, 2.7 mM KCl, 10 mM Na_2_HPO_4_, 2 mM KH_2_PO_4_ pH 7.4) and centrifuged again prior to experiments. For infection, epimastigotes were resuspended in DMEM-2% FBS.

Human foreskin fibroblasts HFF-1 (HFF-1 SCRC-1041) and kidney fibroblasts of *Rhesus* monkey (LLC-MK2) were cultured in an incubator at 37 °C under 5% CO_2_-air atmosphere in Petri dishes or 25 cm^2^ flasks in Dulbecco’s modified Eagle medium (DMEM) from GIBCO (Grand Island, NY, USA) supplemented with 10% FBS (GIBCO, Billings, MT, USA) and antibiotic mixture (SIGMA; St. Louis, MO, USA). At confluence, the cells were detached by trypsinization, collected by centrifugation at 562× *g*, and used for experimentation.

### 4.2. Trypomastigotes Obtention

The protocol of infection was performed as previously described [25]. Briefly, a primary infection was performed where 20,000 HFF-1 cells/mL were cultured in 25 cm^2^ flasks in DMEM-10% FBS. After 24 h, the medium was changed to DMEM supplemented with 2% FBS (DMEM-2% FBS), and the cells were infected with 1 × 10^7^ epimastigotes for 48 h. Thereafter, the medium with unbound epimastigotes was thoroughly mixed, removed, and changed for fresh DMEM-2% FBS, and the cells were incubated as described; the infection was washed daily with the same medium until trypomastigote release at the 4–5th day post infection. Subsequently, 1 × 10^6^ of the released trypomastigotes of the first infection were used for a secondary infection using LLC-MK2 fibroblasts following the same protocol. The trypomastigotes released from this secondary infection were used for the experiments with antichagasic and antidiabetics.

### 4.3. Evaluation of the Mono and Bi-Therapy in the In Vitro Infection

In 24-well tissue culture plates, 2 × 10^4^ HFF-1 cells in 0.5 mL of DMEM + 10% FBS were deposited in each well and incubated at 37 °C for 24 h for adherence. Next, the incubation medium was changed to DMEM + 2% FBS (0.5 mL), and 3 × 10^5^ trypomastigotes were added to infect cells for 2 h at 37 °C. After the incubation, the cells were vigorously washed three times with DMEM + 2% FBS to remove uninternalized trypomastigotes, taking care to preserve the monolayer integrity. Then, the infected cells were incubated in DMEM + 2% FBS for 4–5 days in the absence or presence of BNZ (0, 0.05, 0.1, 0.2, 0.5 µM) and antidiabetics (0, 1, 10, 25, 50 µM) in monotherapy; the drug compounds were purchased from SIGMA. For bi-therapy, the same concentrations of BNZ were used in the presence of 0.5 or 1 µM of antidiabetics. It was previously determined that these antidiabetic concentrations in monotherapy did not significantly affect the HFF-1 viability or the monolayer integrity. In every independent assay, a control of infected cells without BNZ or antidiabetics was always included, which served as no treatment. The number of released mobile trypomastigotes on the 5th day was determined by light microscopy using a Neubauer chamber. The IC_50_ is the drug concentration that decreases by 50% the number of released trypomastigotes.

For HFF-1 IC_50_ evaluation, BNZ and the antidiabetic were varied as described above for the infection cultures, and cell proliferation was determined on the 5th day of growth, and the viability was determined by 0.2% Trypan blue exclusion (Corning Inc.; Corning, NY, USA). Briefly, the cells were washed twice with PBS-EDTA and later trypsinized for detachment. The cell suspension was homogenized with 150 µL of DMEM + 2% FBS, and an aliquot of 50 µL was diluted 1:1 with the solution of Trypan blue. The number of viable cells was counted using a Neubauer chamber under a microscope.

For the IC_50_ calculation, individual data curves were fitted to the dose–response equation:

$$y=A1+(A2-A1)/(1+10^{(logx0-x)\times p}),$$

where A1 < A2; p > 0; bottom asymptote: A1 = 1; top asymptote: A2 = 2; center: logx0 = −1; hill slope: p = 0.2; or logistic equation

$$y=A2+(A1-A2)/{\left(1+x/x0\right)}^{p},$$

where A1 = initial value, A2 = final value, x0 = center and p = power using the Origin 8 Software. The IC_50_ values were manually selected from the curves fit.

### 4.4. Short-Term Antidiabetics Monotherapy in Animals Infected with T. cruzi

Mice of the CD1 strain were infected with Ninoa strain (MHOM/MX/1994/Ninoa) [27,28] following modified protocols previously reported [51,52,53,54]. Briefly, blood trypomastigotes obtained from an infected mouse were diluted with 0.9% saline solution at a density of 1 × 10^5^ trypomastigotes/mL, and 0.1 mL were intraperitoneally inoculated in mice weighing 30–40 g. The lower dose of parasites and higher animal weight allowed better control of the infection and decreased animal mortality. After reaching a parasitemia peak of 22 × 10^6^ blood trypomastigotes/mL (12–18 days post-inoculation), the mice were separated into 5 groups of 3 mice each for drug treatment (for bioethical requirements, the number of animals used was kept at a minimum, and 3 mice per group was sufficient to perform statistical analysis). BNZ and antidiabetic drugs at a concentration of 100 mg/kg weight; doses of 3.16–3.79 mg (depending on the weight of the mice) were administered, diluted in 0.5 mL of vehicle (Arabic gum at 4%, diluted in purified water) using an intragastric tube. Then, 5 µL of blood were immediately extracted from the caudal vein (t = 0), and blood sampling was performed every 2 h up to 8 h. Trypomastigotes were counted using a modified protocol of that reported by Pizzi–Brenner, as reported before [53]. Briefly, the blood sample was placed on an 18 × 18 mm slide, covered with a cover slip, and observed under a microscope at 40× amplification and bright field; mobile trypomastigotes were counted in 15 random fields instead of the 100 random fields used by the Pizzi–Brener method; no statistical difference was observed between the two counting methods. The criterion for evaluating efficacy was the percentage of trypanocidal effect at 8 h compared to time 0.

### 4.5. Effect of the Mono and Bi-Therapy on Thiol Metabolites

Since thiol metabolite determination requires high amounts of biological material, the thiol determination content was performed in epimastigotes of the Queretaro strain. A 0.2 L culture of LIT medium was inoculated with epimastigotes at a density of 0.7 × 10^6^ parasites/mL. The culture was incubated for 72 h at 28 °C, reaching, at the exponential growth phase, a concentration of 3 × 10^6^ parasites/mL. The culture was separated into 8 aliquots of 25 mL, and 1 µM BNZ was added alone or in combination with 1 µM of the antidiabetic drugs. The cultures were incubated for 24 h at 28 °C. Subsequently, 1 mL of each sample was separated for protein determination, and the rest was used for thiol determination. Both samples were centrifuged at 20,817× *g* for 10 min; the pellets were washed twice with PBS. The pellets for protein were resuspended in 100 µL of Lowry’s solution A (2% Na_2_CO_3_, 0.4% NaOH, 0.16% Na-K tartrate, 1% SDS) and stored at −70 °C until protein determination using the Lowry method [55]. The parasite pellets for thiol metabolite determination were immediately lysed by incubation in liquid nitrogen and stored at −70 °C.

Thiol determination was performed as described before [25,31]. Cell lysates were thawed and resuspended in 90 µL of lysis buffer (20 mM Hepes, 1 mM EDTA, 0.15 mM KCl at pH 7), 20 mM DTT was added to each sample, and three cycles of freezing in liquid nitrogen and thawing at 37 °C were performed. The samples were centrifuged at 20,817× *g* for 10 min, and the supernatant was reduced with 2% sodium borohydride (NaBH_4_) and incubated for 10 min on ice. Then, perchloric acid was added at a final concentration of 3%, and the sample was centrifuged at 20,187× *g* for 2 min. The supernatant was filtered through a syringe-driven filter unit (0.45 µm) (Millipore; Carrigtwohill, County Cork, Ireland) and 20 µL of the eluate was injected in a high-performance liquid chromatography (HPLC) apparatus (Waters; Milford, MA, USA). The metabolites were separated in a C18 reverse-phase column (Altima, Columbia, MD, USA) using a mixture of acetonitrile—0.1% trifluoroacetic acid and a flux of 0.5 mL/min. The thiol metabolites were post-column derivatized with 0.1 mM di-thio-bis nitrobenzoic acid (DTNB), and the absorbance was detected at 412 nm with a UV-Vis detector coupled to the HPLC apparatus.

## 5. Conclusions

Antidiabetic drugs that can lower the effective concentration of BNZ to decrease the *T. cruzi* infection were identified. The antidiabetics drugs GBD, GPZ, and GLQ showed trypanocidal activity in initial studies in an animal in vivo model, which should be experimentally analyzed in further detail to delve into the synergy mechanisms. This combinatory therapy may lower the doses of BNZ necessary to eliminate the infection in the host and thus diminish the undesirable adverse side effects of BNZ, which are the main cause of treatment abandonment, particularly in adult patients. Our findings open a new venue to investigate these low-cost, commercially available antidiabetics to improve BNZ treatment against. *T. cruzi* infection. If combinatory therapy is successful, translational studies will be necessary before clinical trial evaluation.

## Figures and Tables

**Table 1:** IC_50_ values of BNZ and antidiabetics in monotherapy and IC_50_ of BNZ in bi-therapy with antidiabetics on Queretaro strain release of trypomastigotes from in vitro infection and on HFF-1 growth.

Treatment	Monotherapy		Bi-Therapy (IC_50_ of BNZ)		
			+1 µM Antidiabetic	+0.5 µM Antidiabetic	+1 µM Antidiabetic
	HFF-1 (*n* = 3)	*T. cruzi* i.v. Infection	HFF-1 (*n* = 2)	*T. cruzi* i.v. Infection (*n* = 8)	*T. cruzi* i.v. Infection (*n* = 8)
BNZ	0.9 ± 0.09	0.26 ± 0.01 (10)	‒		
GBD	12 ± 8	8.4 ± 6.3 (4)	‒		
BNZ + GBD	**‒**		>0.5	0.04 * ± 0.007	0.03 * ± 0.007
GLQ	15 ± 11	7.6 ± 2 (4)	‒		
BNZ + GLQ	‒		0.25	0.05 * ± 0.02	0.04 * ± 0.01
GPZ	35 ±19	14.3 ± 7 (4)	‒		
BNZ + GPZ	‒		>0.5	0.055 * ± 0.02	0.04 * ± 0.01

The IC_50_ concentrations are in µM. i.v.: in vitro. The number of independent experiments is indicated in parentheses. * Two-tailed Student’s *t* test versus BNZ in monotherapy in in vitro infection, *p* < 0.001.

## Data Availability

All data are contained within the article.

## Abbreviations

BNZ, benznidazole; GBD, glyburide; GLQ, gliquidone; GPZ, glipizide; HFF-1, human foreskin fibroblasts 1; Nfx, nifurtimox.
